# A Case of Trigeminal Neuralgia Treated With Combination of Antihistamine, Montelukast, and Corticosteroid Nasal Spray

**DOI:** 10.7759/cureus.12223

**Published:** 2020-12-22

**Authors:** Asna Shahab, Mydah S Hashmi, Zahoor Ahmed, Agha S Haider, Aimen Haider

**Affiliations:** 1 Internal Medicine, Dow International Medical College, Dow University of Health Sciences, Karachi, PAK; 2 Internal Medicine, Army Medical College, Rawalpindi, PAK; 3 Internal Medicine, King Edward Medical University, Mayo Hospital, Lahore, PAK; 4 Internal Medicine/Pulmonology, John Randolph Medical Center, Hopewell, USA; 5 Internal Medicine/Pulmonology, Southside Regional Medical Center, Hopewell, USA; 6 Faculty of Health, York University, North York, CAN

**Keywords:** trigeminal neuralgia, antihistamine, montelukast, corticosteroid nasal spray, refractory pain, allergy

## Abstract

Trigeminal neuralgia (TN) is a nerve disorder of the face associated with excruciating pain that occurs in paroxysms and can be initiated by even mild cutaneous stimuli. Diagnosis of TN is based on the patient’s history and the diagnosis of exclusion. The first-line treatment usually comprises carbamazepine or oxcarbazepine. Herein we present a case of a 47-year-old female, diagnosed with idiopathic TN. Initially, she was commenced on carbamazepine, and later, she was switched to sodium valproate without any noticeable relief. However, she responded to treatment with combination therapy comprising antihistamine, montelukast, and corticosteroid nasal spray. To the best of our knowledge, no such case has been reported previously.

## Introduction

Based on the available clinical data to date, the first-line treatment for trigeminal neuralgia (TN) is carbamazepine and oxcarbazepine. Other drugs such as phenytoin, baclofen, gabapentin, sodium valproate, lamotrigine, and clonazepam can be added if carbamazepine is unresponsive [1,2]. The pathophysiology of neuropathic pain is complex and associated with an increased level of excitatory neurotransmitters and neuropeptides such as histamine, bradykinin, 5-hydroxytryptamine, glutamate [3]. We present the case of a 47-year-old female with right-sided facial pain and mild numbness that readily responded to a combination of an antihistamine, montelukast, and corticosteroid nasal spray.

## Case presentation

A 47-year-old Caucasian female presented with excruciating right-sided facial pain and mild numbness for the past one and a half years. The pain was episodic in nature, occurring every other day, lasting a couple of minutes. The attacks were triggered by the intake of cold drinks and spicy food. The attacks were not associated with any additional symptoms, particularly visual disturbance, headaches, shoulder and pelvic pain, and allergic conditions. Her past medical history was significant for hypothyroidism. On neurological exam, the patient was alert, awake, and oriented, no sensory or motor deficit was noted, and cranial nerves (CN) II-XII were grossly intact. The rest of the rheumatological, otolaryngology, dental, and allergy examination, MRI of the brain and sinuses, and blood and skin allergy workup were unremarkable. Blood work comprised of complete blood count, alanine transaminase, erythrocyte sedimentation rate, C-reactive protein, rheumatoid factor, complement (C3/C4), antinuclear antibody (ANA), double-stranded DNA (ds-DNA), and thyroid-stimulating hormone; all were within the normal range (Table 1).

**Table 1:** Comprehensive blood workup

Blood Work	Results	Reference Range	Unit
Red blood cell count	4.69	4.0-5.1	x E12/L
White blood cell count	7.7	4.0-11.0	x E9/L
Platelets	259	150-400	x E9/L
Eosinophils	0.1	0.0-0.5	x E9/L
Neutrophils	5.2	2.0-7.5	x E9/L
Lymphocytes	2.0	1.0-3.5	x E9/L
Alanine aminotransferase	17	<36	U/L
Erythrocyte sedimentation rate	16	2-30	mm/hr
C-reactive protein	1.9	<5	mg/L
Rheumatoid factor	<10	<14	IU/mL
Complement component 3 (c3)	1.37	0.90-1.80	g/L
Complement component 4 (c4)	0.37	0.15-0.53	g/L
Albumin	46	35-42	g/L
Immunoglobulin class A (IgA)	1.22	0.54-4.17	g/L
Immunoglobulin class M (IgM)	1.32	0.30-2.30	g/L
Immunoglobulin class G (IgG)	9.62	6.00-16.00	g/L
Immunoglobulin class E (IgE)	2.0	<100	kU/L
Thyroid-stimulating hormone	1.64	0.32-4.00	mIU/L
Ferritin	54	5-272	ug/L
Vitamin B12	234	138-652	pmol/L
Antinuclear antibody (ANA)	<1	Negative	Negative
Anti-double-stranded DNA	< 1	<5	IU/mL
Tree mix	Negative	<0.35	KU/L
Dust mite	Negative	<0.35	KU/L
Grass mix	Negative	<0.35	KU/L
Mould mix	Negative	<0.35	KU/L
Chicken feathers	Negative	<0.35	KU/L
Cat and dog dander	Negative	<0.35	KU/L
Food allergy	Negative	<0.35	KU/L

Based on the history, examination, and laboratory findings, a clinical diagnosis of TN was made. The patient was initially started on carbamazepine 100mg two times a day with no relief; eventually, the dose was increased to 1200mg per day for three months with no improvement. Later, she was switched to sodium valproate 200mg per day for one month without any noticeable relief. After the allergy and clinical immunology consultation, though the skin and blood allergy workup were noncontributory, the patient was started on a combination therapy that consisted of tablet rupatadine 10mg, tablet montelukast 10mg, and Avamys nasal spray 27.5mcg two sprays per nostril once daily every day for three months initially. On follow-up, the patient reported significant pain relief, the frequency of the episodes reduced from every other day to 11-12 episodes per year.

## Discussion

TN is defined as paroxysmal attacks of severe shooting, stabbing pain in one or more divisions of the trigeminal nerve that lasts from a fraction of a second to two minutes. The pain is initiated by chewing, light pressure, or gentle stroke on the affected side [4]. The episodes of neuralgia may vary from patient to patient, ranging from nil to 50 per day [5]. In more than half of the patients, there is complete remission between the episodes ranging mostly for a few months [5].

TN can be classical or secondary. The classical TN is due to compression of the trigeminal nerve, mostly by an artery at the level of the nerve root [4]. The most commonly involved artery is the superior cerebellar artery [6]. The nerve root compression results in demyelination, atrophy, and displacement of the nerve. The secondary TN is due to a specific underlying disease such as herpes zoster infection, multiple sclerosis, space-occupying lesion, or tumor at a cerebellopontine angle [4].

TN is a clinical diagnosis. The International Classification of Headache Disorders (ICHD) in its third edition described that the diagnosis of TN requires to fulfill all of the following criteria. There are paroxysms of severe unilateral, non-radiating pain in one or more divisions of the trigeminal nerve stimulated by innocuous stimuli. The pain is severe in intensity and has stabbing, electric shock-like, or shooting characteristics. The duration of pain is variable but ranges from a fraction of a second to two minutes. The pain cannot be explained by any other medical illness in ICHD-3 diagnosis [4]. The incidence of TN increases with age, with a higher incidence in females than males [7].

The first-line treatment for both classical and secondary TN is oral carbamazepine and oxcarbazepine [8]. Add-on therapy with other anticonvulsants, including lamotrigine, pregabalin, gabapentin, and skeletal muscle relaxants such as baclofen, is prescribed to patients with inadequate control of symptoms or emergence of adverse effects at a high dose [8].

A case report of a 48-year-old female by Horton et al. in 1948 showed significant relief in symptoms of TN after administration of first-generation antihistamines. The patient had exacerbation and severe episodes of TN in the spring. The seasonal exacerbation and alleviation of TN symptoms after administration of antihistamine hinted at an underlying allergic etiology [9].

A follow-up clinical study was performed by Hanes on 183 TN patients. Combination therapy of antihistamines with hydrochloric acid and histamine desensitization resulted in complete relief of symptoms in 57.3% of patients and partial relief of symptoms in 11.4% of patients [10]. Currently, a clinical trial on TN patients is being conducted by China Medical University Hospital to assess the therapeutic efficacy of a combination of antihistamine and acupuncture in relieving the neuralgia symptoms. More data is needed to validate the use of combination therapy of antihistamines with montelukast and corticosteroid nasal spray.

## Conclusions

This is one of the first case reports showing improvement of TN symptoms with combination therapy of antihistamines, leukotriene receptor antagonists, and corticosteroids. More clinical studies and trials are needed to study the effectiveness of these drugs in the treatment of TN.
